# The renal metallothionein expression profile is altered in human lupus nephritis

**DOI:** 10.1186/ar2450

**Published:** 2008-07-06

**Authors:** Mikkel Faurschou, Milena Penkowa, Claus Bøgelund Andersen, Henrik Starklint, Søren Jacobsen

**Affiliations:** 1 Department of Rheumatology, The National University Hospital, Rigshospitalet, 9 Blegdamsvej, DK-2100 Copenhagen, Denmark; 2 Section of Neuroprotection, Faculty of Health Sciences, University of Copenhagen, 3 Blegdamsvej, DK-2200 Copenhagen, Denmark; 3 Department of Pathology, The National University Hospital, Rigshospitalet, 9 Blegdamsvej, DK-2100 Copenhagen, Denmark; 4 Department of Pathology, Vejle Hospital, 25 Kabbeltoft, DK-7100 Vejle, Denmark

## Abstract

**Introduction:**

Metallothionein (MT) isoforms I + II are polypeptides with potent antioxidative and anti-inflammatory properties. In healthy kidneys, MT-I+II have been described as intracellular proteins of proximal tubular cells. The aim of the present study was to investigate whether the renal MT-I+II expression profile is altered during lupus nephritis.

**Methods:**

Immunohistochemistry was performed on renal biopsies from 37 patients with lupus nephritis. Four specimens of healthy renal tissue served as controls. Clinicopathological correlation studies and renal survival analyses were performed by means of standard statistical methods.

**Results:**

Proximal tubules displaying epithelial cell MT-I+II depletion in combination with luminal MT-I+II expression were observed in 31 out of 37 of the lupus nephritis specimens, but not in any of the control sections (*P *= 0.006). The tubular MT score, defined as the median number of proximal tubules displaying this MT expression pattern per high-power microscope field (40× magnification), was positively correlated to the creatinine clearance in the lupus nephritis cohort (*P *= 0.01). Furthermore, a tubular MT score below the median value of the cohort emerged as a significant predictor of a poor renal outcome in renal survival analyses. Thus, patients with a tubular MT score < 1.0 had a 6.2-times higher risk of developing end-stage renal disease than patients with a tubular MT score ≥ 1.0 (*P *= 0.03).

**Conclusion:**

Lupus nephritis is associated with significant alterations in renal MT-I+II expression. Our data indicate that important prognostic information can be deduced from the renal MT-I+II expression profile in systemic lupus erythematosus patients with nephritis.

## Introduction

Systemic lupus erythematosus (SLE) is an immune-inflammatory disorder characterized by multiorgan involvement and a chronic relapsing course. Nephritis is a common and serious manifestation of SLE. More than one-half of all SLE patients develop nephritis during their course of illness, and 10% to 25% of these patients progress to end-stage renal disease (ESRD) despite immunosuppressive treatment [1-4]. A central pathogenic aspect of lupus nephritis is the deposition of immune complexes within glomeruli and tubules. Subsequent inflammatory processes involving complement activation, macrophage recruitment, and generation of reactive oxygen species cause glomerular damage, increased glomerular permeability, and proteinuria. Sustained glomerular and interstitial inflammation stimulates fibrogenesis, eventually leading to renal scarring and compromised renal function [1,5].

Metallothioneins (MTs) are nonenzymatic polypeptides of 6 to 7 kDa that bind heavy metals with high affinity and possess a range of anti-inflammatory properties [6]. Due to a high content of cysteines, MTs are potent antioxidants and provide protection against reactive oxygen species-induced cellular damage during experimental inflammation [7-10]. In addition, MTs have been shown to reduce inflammation through interference with activation of cytotoxic T cells and B cells [11,12]. Four major MT subfamilies exist in mammals (MT-I, MT-II, MT-III, and MT-IV). Human kidneys express MT-I and MT-II, which have been described as intracellular proteins of proximal tubular cells [13,14].

The aim of the present study was to investigate whether the pathogenesis of lupus nephritis involves an altered pattern of renal MT-I+II expression. To our knowledge, this question has not been addressed before. We demonstrate that lupus nephritis is associated with significant alterations in the tubular MT-I+II expression profile. Furthermore, our data indicate that important prognostic information can be deduced from the renal MT-I+II expression pattern in patients with lupus nephritis.

## Materials and methods

### Patients and biopsy specimens

Formalin-fixed, paraffin-embedded renal biopsies were obtained from patients of a previously described historic lupus nephritis cohort [4]. All patients were diagnosed with lupus nephritis during the period 1975 to 1995 and met the classification criteria of SLE defined by the American College of Rheumatology [15]. Tissue suitable for experimental immunohistochemistry could be retrieved for 37 patients. These biopsies were kindly made available from the Departments of Pathology at Odense University Hospital, Denmark, at Aarhus University Hospital, Denmark, at Herlev University Hospital, Denmark, and at The National University Hospital, Rigshospitalet, Copenhagen, Denmark. All biopsies had been taken for diagnostic purposes, and no patients had previously been diagnosed with lupus nephritis. Samples of normal kidney tissue obtained from four patients with renal cell carcinoma served as control specimens. In addition, renal biopsies from five patients suffering from antineutrophil cytoplasmic autoantibody-associated systemic vasculitis (AASV) with glomerulonephritis were retrieved (two patients with Wegener's granulomatosis, three patients with microscopic polyangiitis).

The following data were registered at the time of biopsy for all SLE patients: sex, age at time of biopsy, age at time of SLE diagnosis, date of first renal biopsy, date of onset of nephritis symptoms (defined as the date of the first observation of persistent proteinuria (> 0.5 g/day), hematuria, and/or cellular casts), serum creatinine, creatinine clearance, serum albumin, blood pressure, level of 24-hour urinary protein excretion, a clinical disease activity score (representing a modified version of the European Consensus Lupus Activity Measurement [16], using only the clinical components with unchanged scoring weights), and a urinary sediment analysis. The urinary sediment was considered indicative of active renal disease if analysis showed cellular or granular casts or > 5 erythrocytes per high-power field. Treatment with cyclophosphamide, azathioprine, and high-dose cortico steroids was recorded. The time from kidney biopsy to development of ESRD (defined as the need for chronic dialysis or renal transplantation) was known in all cases. The study baseline was defined as the date of first renal biopsy. Patients were followed until death or until the end of 1995. No patients were lost to follow-up.

### Standard histopathological analyses

All SLE biopsies were examined (by HS) and classified according to the World Health Organization (WHO) classification criteria for lupus nephritis [17]. Activity index scores and chronicity index scores were calculated using the scoring system developed by the US National Institutes of Health [18]. According to this system, active glomerular lesions include cellular proliferation, fibrinoid necrosis/karyorrhexis, cellular crescents, hyaline thrombi/wire loops, and leukocyte infiltration – while mononuclear cell infiltration is considered an active tubulointerstitial abnormality. Chronic glomerular lesions encompass glomerular sclerosis and fibrous crescents, while chronic tubulointerstitial changes include interstitial fibrosis and tubular atrophy. Each variable is scored from zero to three, weighting fibrinoid necrosis/karyorrhexis and cellular crescents by a factor of two. The biopsies were evaluated without knowledge of clinical data.

### Immunohistochemistry

Paraffin-embedded renal biopsies were cut into 5 μm-thick sections and were processed for immunohistochemistry as previously described [19,20]. In brief, sections were incubated overnight at 4°C with rabbit anti-MT-I+II, diluted 1:500 [20-23]. The primary antibody was detected using biotinylated mouse antirabbit IgG (1:400, code B3275; Sigma-Aldrich, Copenhagen, Denmark), followed by streptavidin–biotin–peroxidase complex (StreptABComplex/HRP, code K377; Dakopatts, Glostrup, Denmark) prepared according to the manufacturer's recommended dilution. These secondary and tertiary steps were performed at room temperature for 30 minutes. The immunoreaction was visualized using 0.015% H_2_O_2 _in 3.3-diaminobenzidine-tetrahydrochloride (DAB)/Tris-buffered saline (TBS: 0.05 M Tris, 0.15 M NaCl, pH 7.4) for 10 minutes at room temperature. To evaluate the extent of nonspecific binding in the immunohistochemical experiments, control sections were incubated in the absence of primary antibody.

### Evaluation of immunohistochemically stained slides

To obtain an overall impression of the MT-I+II expression patterns, anti-MT-I+II-stained sections were first screened by two investigators (MF and MP). In the healthy kidney specimens, a strong MT-I+II staining reaction was observed in the cytoplasm of proximal tubular cells. Furthermore, cytoplasmic MT-I+II staining was seen in the epithelial cells of some collecting tubules as described below. In the lupus nephritis specimens, MT-I+II staining was also found in the proximal renal tubules. In a large proportion of these specimens, however, some proximal tubules displayed intense luminal MT-I+II reactivity in combination with absent or significantly reduced epithelial cell staining. The number of cross-sectioned proximal tubules displaying the latter pattern of anti-MT-I+II reactivity was determined in a sequence of 3 to 60 consecutive high power fields (40× magnification), depending on the size of the individual biopsy. The only field adjustments were made to avoid large vessels and glomeruli.

For semiquantitative analyses, each cross-sectioned proximal tubule displaying significant luminal MT-I+II reactivity in combination with reduced/absent staining of the lining epithelial cells was scored one, while cross-sectioned proximal tubules in which the MT-I+II staining was observed exclusively/predominantly in the cytoplasm of the epithelial cells were scored zero. As a conservative approach, tubules that did not clearly display one of these morphologies were also scored zero. The tubular MT score of the individual biopsy specimens was defined as the median score obtained according to this scoring system per visual field at 40× magnification.

Most of the lupus nephritis biopsies consisted mainly of cortical renal tissue. Consequently, the MT-I+II expression in collecting tubules could not be systematically examined in these specimens. In all biopsies, glomerular cross-sections were also evaluated for MT-I+II reactivity, and the median number of MT-I+II-positive cells was determined.

All biopsies were scored by the same investigator (MF) and were analyzed without knowledge of clinical or standard histopathological data. The scorings were subsequently validated by another investigator (CBA).

### Visualization

Sections were examined on an Axioplan plus light microscope (Zeiss, Göttingen, Germany). Images were recorded using a digital camera (Coolsnap 1.2; RS Photometrics, Tucson, AZ, USA).

### Statistics

The cumulative incidence of ESRD was calculated using life tables and the Kaplan–Meier method. The equality of cumulative incidence curves was tested by the log-rank test. Event rates were related to the total number of person-years of observation, and rate ratios of event rates were calculated in stratified analyses. Comparison of continuous data was performed using the Mann–Whitney rank-sum test. Spearman's rank correlation test was used in correlation studies.

In all analyses, *P *< 0.05 (two-tailed) was considered statistically significant. The statistical analyses were performed on a computer using SPSS version 9.0 for Windows (SPSS, Chicago, IL, USA).

### Ethics

The present study was approved by the local ethics committee (journal number 01 310445) and by the Danish Data Protection Agency (journal number 2006060030A).

## Results

### Basic clinical and histopathological findings

Clinical data of the 37 SLE patients are summarized in Table 1. Thirty-two of the 37 patients were women. Seven patients progressed to ESRD during 161 patient-years of observation, and the cumulative incidence of ESRD after 1 year, 5 years, and 10 years was 6%, 25%, and 36%, respectively. Twenty-two patients (59%) received treatment with cyclophosphamide during their course of illness, a similar number (59%) of patients were treated with azathioprine, and five (13%) patients were subjected to treatment with high-dose corticosteroids only. In all cases, immunosuppressive treatment was started or intensified within 1 month following renal biopsy, and no patients received intensive immunosuppressive treatment before renal biopsy was performed.

**Table 1:** Clinical data of 37 patients with lupus nephritis at the time of first renal biopsy

	Number of patients	Median	Lower quartile	Upper quartile
Age (years)	37	29.6	19.6	46.5
Duration of systemic lupus erythematosus before biopsy (years)	37	0.4	0.0	7.6
Duration of follow-up (years)^a^	37	5.0	1.1	8.0
Creatinine clearance (% of expected)	37	77	60	100
Serum albumin (μmol/l)	37	411	304	498
24-hour urinary protein excretion (g)	37	4.9	2.1	15.1
Systolic blood pressure (mmHg)	36	130	116	160
Diastolic blood pressure (mmHg)	36	90	71	90

^a^Duration of follow-up after first renal biopsy.

Four patients (11%) presented with WHO class II glomerulonephritis, three patients (8%) with WHO class III glomerulonephritis, 27 patients (73%) displayed WHO class IV histopathology, and three patients were diagnosed with WHO class V glomerulonephritis (8%). The median histological activity index score in the cohort was 5.0 (range = 0 to 14), while the median chronicity index score was 1.0 (range = 0 to 9). The median activity index score was significantly higher for patients presenting with WHO class IV glomerulonephritis (median activity index score = 7.0 (range = 2 to 14)) than for patients with other WHO classes of nephritis (median activity index score = 1.5 (range = 0 to 4); *P *< 0.0001). In contrast, the median chronicity index score was not significantly higher in patients with WHO class IV nephritis (median chronicity index score = 1.0 (range = 0 to 9)) than in patients displaying other renal histopathologies (median chronicity index score = 0.0 (range = 0 to 3); *P *= 0.21).

### Metallothionein expression in healthy kidneys, lupus nephritis, and AASV-associated glomerulonephritis

**Figure 1:**
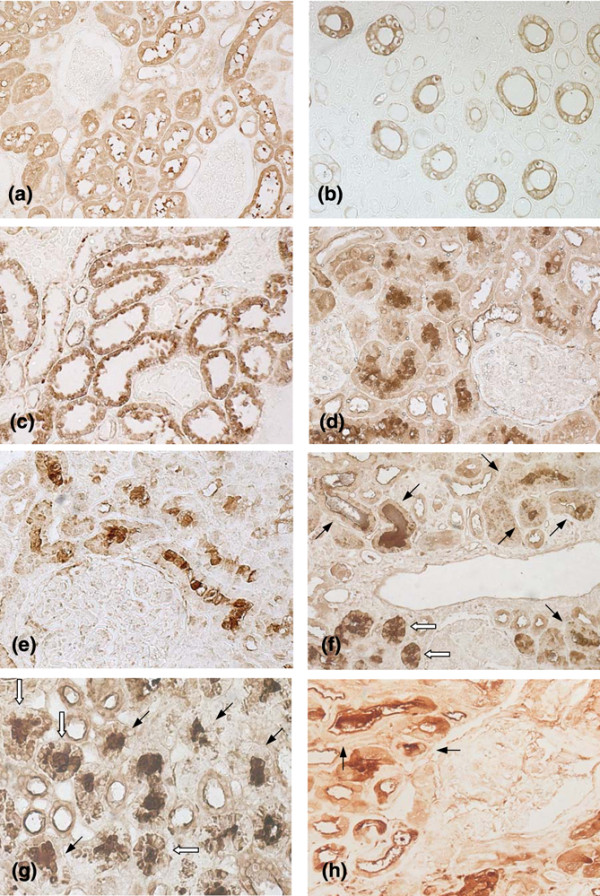
Localization of metallothionein (MT) isoforms I + II in healthy kidneys and in lupus nephritis. Representative results of immunohistochemical analyses. **(a) **Healthy renal tissue. CytoplasmicMT-I+II staining is seen in proximal tubular cells. Concurrent MT-I+II staining is frequently observed in material radiating from the epithelial brush border into the tubular lumen. No staining is observed in glomeruli. **(b) **Inner medullary zone of healthy kidney. MT-I+II staining is seen in the epithelium of the collecting tubules but not in cross-sectioned thin segments of Henle's loop. **(c) **Renal tissue from patient with lupus nephritis and a tubular MT score of zero as defined in the text. Cytoplasmic MT-I+II staining is observed in proximal tubular cells. **(d) **Renal tissue from another patient with lupus nephritis. Intense luminal MT-I+II staining with concomitant epithelial cell MT-I+II depletion is observed in several cross-sectioned proximal tubules. Glomeruli are MT-I+II-negative. **(e) **Lupus nephritis. Same findings as in (d). **(f) **Lupus nephritis. Some proximal tubules display pronounced luminal MT-I+II staining in combination with MT-I+II depletion of the lining epithelial cells (black arrows). In other tubules, some epithelial cells display cytoplasmic MT-I+II staining (white arrows). **(g) **Lupus nephritis. Several cross-sectioned proximal tubules with predominantly luminal MT-I+II staining are shown. As in (f), some tubules display MT-I+II depletion of the epithelium (black arrows). In other tubules, cytoplasmic MT-I+II staining is seen in a proportion of the epithelial cells (white arrows). **(h) **Pauci-immune glomerulonephritis in Wegener's granulomatosis. Two tubules with a predominantly luminal MT-I+II expression pattern are marked (arrows).

Anti-MT-I+II staining of control specimens confirmed that MT-I+II are expressed in the cytoplasm of proximal tubular cells under normal conditions (Figure 1a) [13,14]. A concurrent staining reaction was observed frequently in material radiating from the brush border of the tubular epithelium into the tubular lumen. In all control specimens, however, the strongest anti-MT-I+II reactivity was consistently observed in the tubular epithelium, and no proximal tubules displayed significant luminal anti-MT-I+II reactivity in combination with reduced or absent epithelial cell staining. These findings are similar to observations previously reported by Nartey and colleagues [13].

Three of the four control sections contained enough medullary renal tissue for an examination of medullary structures to be performed. As a novel finding [13,14], we detected significant epithelial cell MT-I+II staining in the collecting tubules of these specimens (Figure 1b). The MT-I+II staining observed in collecting tubules was frequently weaker than the staining reaction observed in proximal tubules, and some cross-sectioned collecting tubules were MT-I+II-negative. Moreover, while the epithelial cells of some collecting tubules displayed a homogeneous cytoplasmic MT-I+II staining reaction (Figure 1b), the MT-I+II staining observed in other collecting tubules was confined to the cytoplasm adjacent to the luminal membrane (data not shown). Glomeruli and other segments of the nephron were consistently MT-I+II-negative. Furthermore, no significant MT-I+II staining was observed in the renal interstitium (Figure 1a, b).

In six out of the 37 lupus nephritis specimens, intense cytoplasmic MT-I+II staining was observed in the epithelial cells lining the proximal tubules (Figure 1c). The MT-I+II staining pattern observed in these biopsies did not differ significantly from the pattern observed in the control specimens. In the remaining lupus nephritis biopsies, we noted the presence of proximal tubules displaying low to absent epithelial MT-I+II staining in combination with strong staining of the tubular lumen. In these tubules, the MTs seemed to be present in the tubular lumen as extracellular proteins (Figures 1d to 1g).

Only nine (24%) of the lupus nephritis biopsies contained significant amounts of medullary renal tissue. In these specimens, collecting tubules displayed the same epithelial MT-I+II expression pattern as observed in the control biopsies (data not shown). Glomeruli and interstitial structures were MT-I+II-negative in all lupus nephritis specimens (Figure 1d to 1f).

To evaluate whether similar changes in renal MT-I+II expression occur in the setting of other inflammatory nephropathies, we finally studied the tubular MT-I+II expression pattern in biopsies from five patients with AASV and glomerulonephritis. In four out of five of these biopsies, we observed the occasional presence of tubules displaying the same alterations in MT-I+II expression as described above (Figure 1h).

### Metallothionein scores and correlation studies

The median tubular MT score was significantly higher in the lupus nephritis specimens (median tubular MT score of the 37 lupus nephritis biopsies = 1.0 (range = 0.0 to 6.8)) than in the control specimens (median tubular MT score of the four control specimens = 0.0 (range = 0); *P *= 0.006) and in the AASV biopsies (median tubular MT score of the five AASV specimens = 0.16 (range = 0.0 to 0.27); *P *= 0.04). Among the SLE patients, the median tubular MT score tended to be lower for patients with WHO class IV nephritis than for patients presenting with other WHO histopathologies (median tubular MT score for 27 patients with WHO class IV nephritis = 0.8 (range = 0.0 to 6.8); median tubular MT score for the 10 other patients = 1.4 (range = 0.1 to 3.6)). This difference was not, however, statistically significant (*P *= 0.07).

**Figure 2:**
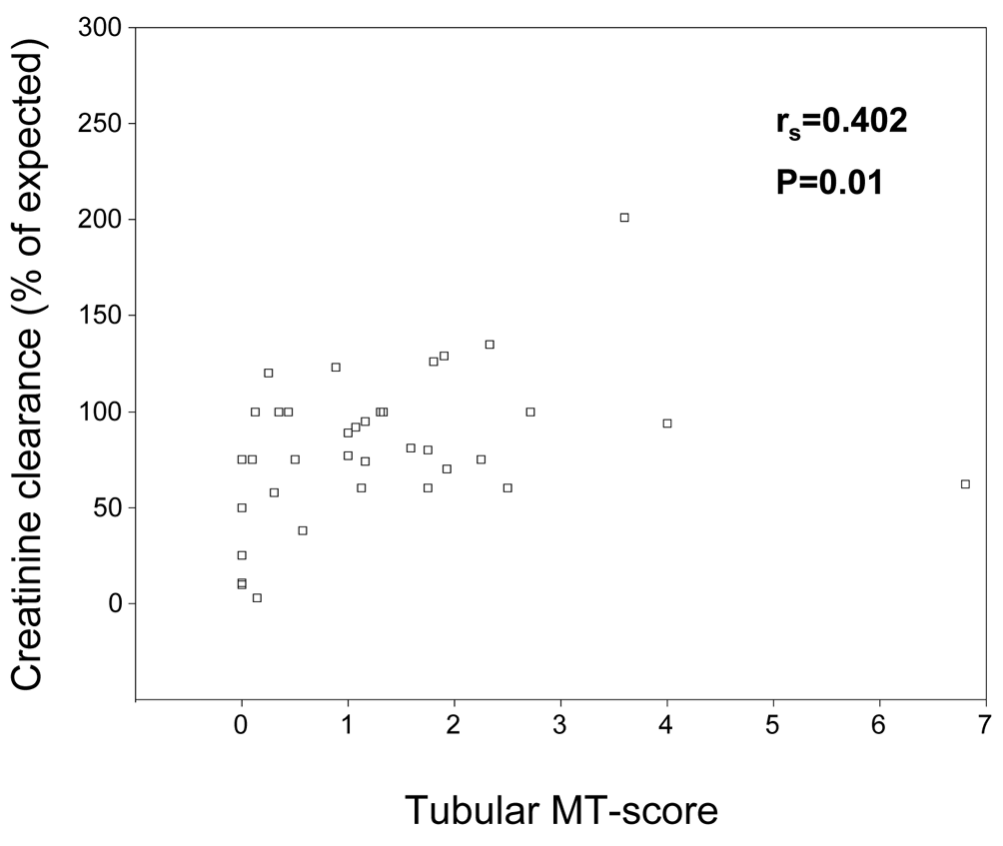
Tubular metallothionein score versus creatinine clearance. Scatterplot of tubular metallothionein (MT) score versus creatinine clearance in a cohort of 37 patients with lupus nephritis. A statistically significant, positive correlation was found between these variables using Spearman's rank correlation test. *r*_s_, Spearman's rank correlation coefficient.

To examine potential associations between the tubular MT score and standard clinicopathological variables in the lupus nephritis cohort, we investigated whether the subset of lupus patients with tubular MT score = 0 (*n* = 6) differed from patients with tubular MT score > 0 (*n* = 31) with regards to baseline levels of selected variables reflecting renal function, including creatinine clearance, level of 24-hour urinary protein excretion, serum albumin, activity index score, chronicity index score, and blood pressure (Table 2). This analysis showed that patients presenting with a tubular MT score of zero had a significantly lower median creatinine clearance than the rest of the patients. To further analyze the association between the tubular MT score and the creatinine clearance, we examined whether these variables were correlated to each other in the lupus cohort other using Spearman's rank correlation test. In this analysis, a statistically significant, positive correlation was found between the tubular MT score and the creatinine clearance (Figure 2).

**Table 2:** Median values of selected clinical variables in a cohort of 37 patients with lupus nephritis

Variable	MT score = 0	MT score > 0	*P *value^a^
Creatinine clearance (% of expected)	37.5 (10.0 to 75.0)	89.0 (3.0 to 201.0)	0.006
24-hour urinary protein excretion (g)	7.6 (2.7 to 21.8)	4.8 (0.2 to 31.9)	0.3
Serum albumin (μmol/l)	265.5 (60 to 480)	420 (150 to 615)	0.06
Activity index score	3.0 (2 to 10)	5.0 (0 to 14)	0.8
Chronicity index score	2.0 (0 to 7)	1.0 (0 to 9)	0.5
Systolic blood pressure (mmHg)	125 (105 to 200)	135 (100 to 200)	0.5
Diastolic blood pressure (mmHg)	85 (70 to 110)	90 (60 to 160)	0.9

Data presented as the median (range). Patients with a tubular metallothionein (MT) score = 0 (*n* = 6) versus patients with a tubular MT score > 0 (*n* = 31). MT score as defined in text. ^a^Mann–Whitney rank-sum test.

### Risk factors for end-stage renal disease

The patients in the present cohort were part of a larger cohort of lupus nephritis patients [4]. In the original cohort, several clinical and histological findings emerged as significant univariate predictors of ESRD (duration of nephritis symptoms prior to biopsy ≥ 6 months, serum creatinine ≥ 140 μmol/l, WHO class IV nephritis, chronicity index score ≥ 4, glomerular sclerosis, fibrous crescents, interstitial fibrosis, and tubular atrophy).

In the much smaller cohort of the present study, only a chronicity index score ≥ 4 and a index score for interstitial fibrosis ≥ 2, i.e., histological findings reflecting chronic renal damage, were identified as significant risk factors for ESRD in dichotomized analyses of standard clinical and histological variables (Table 3). The following clinicopathological variables were not associated with an increased risk of progression towards ESRD: sex; age at time of biopsy; duration of nephritis symptoms prior to biopsy ≥ 6 months; treatment without cyclophosphamide; treatment without azathioprine; modified European Consensus Lupus Activity Measurement score ≥ 4; hypercreatininaemia (serum creatinine ≥ 140 μmol/l); hypoalbuminaemia (serum albumin < 300 μmol/l); active urinary sediment, 24-hour urinary protein excretion ≥ 10 g; systolic blood pressure > 160 mmHg; diastolic blood pressure > 110 mmHg; tubular MT score = 0; WHO class IV histopathology; high activity index scores (cutoff levels tested: ≥ 5, ≥ 7, ≥ 10); and index scores for glomerular sclerosis, fibrous crescents, and tubular atrophy ≥ 2.

**Table 3:** Significant predictors of end-stage renal disease (ESRD) in a cohort of 37 patients with lupus nephritis

Variable	Number of ESRD patients/total number of patients	Patient-years	ESRD rate/1,000 patient-years	Rate ratio	*P *value^a^
Tubular MT score^b^					
< 1.0	6/16	79	75.9	6.2	0.03
≥ 1.0	1/21	82	12.1	1.0	
Chronicity index score					
< 4	4/30	137	29.2	1	0.04
≥ 4	3/7	24	125	4.2	
Interstitial fibrosis					
< 2	4/31	143.5	27.8	1	0.008
≥ 2	3/6	17.5	171.4	6.1	

^a^Log-rank test. ^b^Metallothionein score as defined in the text.

**Figure 3:**
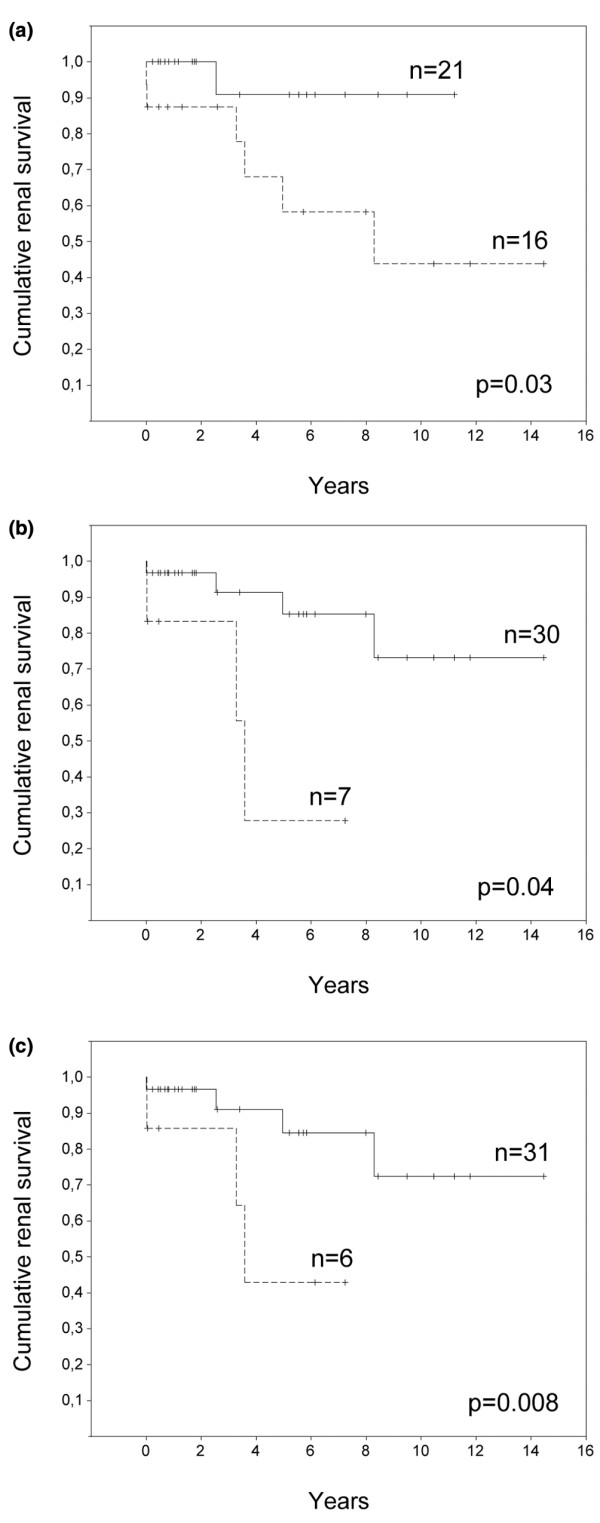
Cumulative renal survival curves for lupus nephritis patients. Cumulative renal survival curves for 37 lupus nephritis patients in analyses stratified as described. **(a) **Patients with a tubular metallothionein (MT) score ≥ 1.0 (solid line) versus patients with a tubular MT score < 1.0 (dashed line). **(b) **Patients with a chronicity index score < 4.0 (solid line) versus patients with a chronicity index score ≥ 4.0 (dashed line). **(c) **Patients with a score for interstitial fibrosis < 2.0 (solid line) versus patients with a score for interstitial fibrosis ≥ 2.0 (dashed line). Kaplan–Meier analyses with the log-rank test.

Interestingly, a tubular MT score below the median value of the cohort emerged as the most powerful predictor of a poor renal outcome among the variables tested. Thus, patients with a tubular MT score < 1.0 had a 6.2-times higher risk of progression towards ESRD than the rest of the patients (Table 3). Cumulative renal survival curves are shown in Figure 3.

## Discussion

Proximal tubules are known to elicit a variety of responses to protein overload during proteinuric nephropathies. Exposure to high protein concentrations stimulates proximal tubular cells to secrete proinflammatory mediators, including monocyte chemoattractant protein 1 [24], regulation upon activation, normal T-cell expressed and secreted (RANTES) [25], IL-8 [26], complement factor C3 [27], and transforming growth factor beta [28]. During lupus nephritis, proximal tubules have been shown to alter their expression of several molecules involved in inflammatory processes, including intracellular adhesion molecule 1, CD40, and Toll-like receptor 9 [29,30].

In the present study, we demonstrate that proximal tubules exposed to the inflammatory and proteinuric conditions associated with lupus nephritis also display a significantly altered expression pattern of MT-I+II. By immunohistochemical analyses, we noted a relatively high frequency of cross-sectioned proximal tubules showing luminal MT-I+II expression with concomitant epithelial cell MT-I+II depletion in kidney biopsies from lupus nephritis patients. Similar changes in renal MT-I+II expression were not observed in biopsies of healthy renal tissue. Importantly, the tubular MT score, reflecting the incidence of proximal tubules displaying these changes in MT-I+II expression, was positively correlated to the creatinine clearance in the SLE cohort, and lupus patients with a tubular MT score of zero presented with a significantly lower median creatinine clearance than the rest of the patients. Moreover, a tubular MT score < 1.0 was identified as a strong predictor of ESRD in renal survival analyses. A low tubular MT score was therefore associated with impaired renal function at the time of first kidney biopsy and with an increased risk of a poor renal outcome in the present SLE cohort, indicating that renal MTs exert important renoprotective functions in the lumen of proximal tubules during lupus nephritis.

The present study was based on a historic cohort of patients and has several limitations. Due to the retrospective nature of the investigations, we were unable to obtain patient material such as serum, urine, or additional renal tissue for further experimental analyses. We were therefore prevented from investigating whether the tubular MT score was correlated to the level of urinary MT-I+II excretion or to the serum concentration of these MTs. Consequently, the present study does not elucidate whether the urinary MT concentration can be used as a biomarker for active nephritis in SLE patients in the same way as previously demonstrated for molecules such as vascular cell adhesion molecule 1, P-selectin, soluble TNF receptor 1 and chemokine (C–X–C motif) ligand 16 [31,32]. Furthermore, we were unable to investigate the origin of the MTs observed in the lumen of proximal tubules during lupus nephritis. In the examined nephritis specimens, luminal MT-I+II expression was consistently associated with MT-I+II depletion of the adjacent tubular epithelium, but not with enhanced MT-I+II expression in other nephron segments or in the renal interstitium. Moreover, light microscopic analyses suggested that MT-I+II are present in the lumen of proximal tubules as extracellular proteins. Together, these findings strongly indicate that the MTs observed in the lumen of proximal tubules were, at least in part, secreted from adjacent tubular cells and not simply derived from plasma via glomerular ultrafiltration. Further studies are needed, however, to determine whether proximal tubular cells secrete MT-I+II to the tubular lumen in the setting of lupus nephritis – and, if so, to identify the stimuli that trigger the secretion.

Intriguingly, our analyses demonstrate that proximal tubules do not always display intraluminal MT-I+II immunostaining during lupus nephritis, suggesting that this alteration in renal MT distribution requires or can be blocked by as yet undefined stimuli. To this end, it could be hypothesized that proximal tubular cells secrete MTs in response to the presence of harmful substances delivered to the tubular lumen from the inflamed glomerulus, but that this secretion can be inhibited by other intracellular or extracellular signals associated with renal inflammation. Although the present study was not designed to investigate the extent to which similar changes in renal MT expression occur during other inflammatory nephropathies, our analyses indicate that the same morphological tubular alterations are seen in AASV glomerulonephritis. This observation suggests that the mechanisms responsible for inducing the observed alterations in the tubular MT expression profile are not specific for lupus nephritis, but are probably evoked by renal inflammation *per se*.

A range of histological features has been shown to influence the prognosis in lupus nephritis. Frequently reported histological predictors of a poor renal outcome include WHO class IV histopathology [4,33,34], high activity index scores and chronicity index scores [4,18,35], high index scores for cellular crescents [35,36], glomerular sclerosis [4,35], interstitial fibrosis [4,35-37], and tubular atrophy [4,35]. Thus, histological variables identified as risk factors of ESRD typically reflect either active inflammation or the late-stage, degenerative consequences of inflammation. Here, we provide evidence that the renal expression pattern of MT-I+II, i.e., proteins with potent anti-inflammatory and antioxidative properties, also carries prognostic information in lupus nephritis. The findings of our study add to the growing amount of experimental data suggesting that MT-I+II exert important functions not only as intracellular proteins, as previously suggested [38,39], but also after secretion from MT-producing cells [9,40].

Furthermore, our study raises a number of new questions. Based on the known functional repertoire of MT-I+II, it is tempting to speculate that MT-I+II protect the proximal tubular epithelium from reactive oxygen species and other cytotoxic substances after secretion to the tubular lumen as outlined above. Nevertheless, the mechanisms by which these MTs provide renoprotection during lupus nephritis remain to be defined. It also remains to be clarified to what extent the observed changes in tubular MT-I+II expression are evoked in the setting of other inflammatory nephropathies. Finally, from a therapeutic perspective it would be of great interest to investigate whether treatment with exogenous MT-I+II could modify the course of lupus nephritis in animal models.

## Conclusion

Our study provides the first description of renal MT-I+II expression during lupus nephritis. Compared with the MT-I+II expression pattern observed in healthy renal tissue, characteristic alterations in the tubular MT expression profile were found in most of the examined lupus nephritis specimens. In renal survival analyses, these alterations were found to be of prognostic significance.

## Abbreviations

AASV = antineutrophil cytoplasmic autoantibody-associated systemic vasculitis; ESRD = end-stage renal disease; IL = interleukin; MT = metallothionein; SLE = systemic lupus erythematosus; TNF = tumor necrosis factor; WHO = World Health Organization.

## Competing interests

The authors declare that they have no competing interests.

## Authors' contributions

MF, MP, and SJ designed the study. MF analyzed the immunohistochemically stained slides, performed the data analysis, and prepared the manuscript. MP performed the immunohistochemistry assays and contributed to the analysis of the stained slides. CBA contributed to the analysis of the stained slides and validated the tubular MT scorings. HS performed the standard histopathological analyses. SJ provided the clinical data of the cohort and contributed to the data analysis. All authors read and approved the final manuscript.
